# Conventional colon adenomas harbor various disturbances in microsatellite stability and contain micro-serrated foci with microsatellite instability

**DOI:** 10.1371/journal.pone.0172381

**Published:** 2017-02-24

**Authors:** Piotr Lewitowicz, Stanislaw Gluszek, Dorota Koziel, Agata Horecka-Lewitowicz, Magdalena Chrapek, Przemyslaw Wolak, Justyna Klusek, Anna Nasierowska-Guttmejer

**Affiliations:** 1 Department of Pathology, Faculty of Medicine and Health Sciences, Jan Kochanowski University, Kielce, Poland; 2 Department of Surgery and Surgical Nursing, Faculty of Medicine and Health Sciences, Jan Kochanowski University, Kielce, Poland; 3 Department of Public Health, Faculty of Medicine and Health Sciences, Jan Kochanowski University, Kielce, Poland; 4 Department of Probability Theory and Statistics, Institute of Mathematics, The Faculty of Mathematics and Natural Sciences, Jan Kochanowski University, Kielce, Poland; 5 Department of Anatomy, Faculty of Medicine and Health Sciences, Jan Kochanowski University, Kielce, Poland; Howard University, UNITED STATES

## Abstract

**Introduction:**

Colorectal cancer belongs to the most frequent occurring malignancies. A prediction of the clinical outcome and appropriate choice of neoadjuvant chemotherapy needs personalized insight to the main driving pathways. Because most CRCs have polyps as progenitor lesions, studying the pathways driving to adenomagenesis is no less important.

**Goals:**

Our purpose was the evaluation of microsatellite stability status within conventional colon adenomas and also β-catenin, BRAFV600E and p53 contribution.

**Material and methods:**

The cohort included 101 cases of typical colon adenomas with high grade epithelial dysplasia according to WHO. An immunohistochemistry method was used for the depiction of the expression of targeted proteins, as also their heterogeneity.

**Results:**

Generally, we noted a 10% frequency of MSI events where MSI-H reached a 5% share occurred within the left colon and rectal polyps. β-catenin nuclear overexpression was noted with a 70% frequency and p53 with close to a 24% frequency. In addition, we found a presence of micro-serration foci more often within tubular adenomas, where focal MSI took place more often. Our results indicate that MSI events occur more often than had been theorized earlier. It results in tumour heterogeneity, more complex underlying pathways and finally ontogenetic molecular-diversity of tumours besides similar occurring histopathological features.

## Introduction

Despite marked progress in the area of molecular alterations underlying colorectal cancer (CRC) and widely implemented screening programs, mostly in western countries, CRCs are still one of the most frequently occurring human malignancies [1–3]. In order for prevention programs to be efficient, one needs to understand the key-factors leading to the occurrence of CRC. This is a unique type of cancer, where precancerous lesions are well defined and known as adenomatous or serrated polyps. In general, most types of colon adenomatous lesions are unquestionably and commonly accepted lesions of cancer genesis. It might seem that all has been said about colon polyps, but the broad heterogeneity of CRC also indicates many signaling pathways leading to the development of adenomas. Understanding cancer should be preceded by as far as possible understanding polyps and all alterations involved in their genesis.

All of this began with the discovery of the APC gene mutation and the presentation of the canonical adenoma-carcinoma sequence. Subsequently, additional information about the contribution of the whole Wnt pathway, TP53, KRAS and BRAF mutations provided an insight to the pathogenesis of polyps. It is estimated, similarly to CRCs, that the frequency of the chromosomal instability pathway (CIN) covers about 80–90% of all adenomatous polyps [1–3]. Mistuning of Wnt signaling route results in the loss or attenuation of APC–GSK3β complex and improves β-catenin quantity and also various pro-proliferative outputs. Their downstream influence is mainly towards cell-cycle regulation or apoptosis. More recently attention has been focused on microsatellite instability (MSI). The prototype of this model was described by Lynch as the molecular alterations within DNA mishmash repair (MMR) genes leading to non-polyposis colorectal cancer (HNPCC) in 1988 [4]. Currently, this pathway is becoming more and more valuable in planning neoadjuvant therapy in cases of CRC. The flagship Bethesda-panel microsatellite loci include BAT25, BAT26, D2S123, D5346 and D1S250, although screening is more frequently based on the immunohistochemical expression of the following: MLH1, MSH2, MSH6, and PMS2. The MSI contribution to CRC is estimated at about 15–20%, where the majority is caused by inherited mutations. Moreover, it is believed that the distinct histopathogical features of MSI, such as the mucinous cancer type or florid lymphoid infiltration, predict a better patients’ clinical outcome [3]. A lot of time and effort has been invested into clarifying the differences of survival rate, treatment cost and neo-adjuvant treatment efficiency within MSS and MSI patients. Although there are visible attempts to routinely assign MSS/MSI status, solid, hard guidelines have not been implemented yet. The next recently added pathomechanism of developing adenomas with the characteristic serrated features is the CpG methylation pathway(CIMP). Both the characteristic histopathological pattern and the right-side location of the flat or sessile mucosal lesions are typical for this path and currently this detached group of unique polyps is called serrated adenomas/polyps (SSA/SSP). An interesting fact is that a very similar pattern as the saw-tooth like appearance of the glands is also the pattern of hyperplastic polyps(HPs), which are mainly located in the distal colon and rectum. Notwithstanding the fact that the morphological appearance is very similar, the driving pathway in HPs is often caused by the BRAF mutation. What the driving engineering pathways of various morphological patterns within traditional adenomas as tubular, villous or mixed patterns are, have not been completely illustrated to date. The incidence of the BRAF mutation, especially within the V600E loci, is estimated at about 10%, but within the serrated-pathway the frequency ratio is close to 60% [1, 3, 5]. Because both the BRAF mutation as well the CIMP can result in a serrated appearance, the heterogeneity of the various pathways leading to that is interesting. Hypermethylation could often involve MLH-1, and the rarer MSH-2 gene and consequently lead to a ‘secondary’ MSI status. This proves that serration might be the result of equally a BRAF mutation, MSI, epigenetic alterations or the overlapping of various pathways. The recently discovered additional CRC pathway is the MUTYH polyposis (MAP). The MUTYH gene is involved in base excision repair following oxidative damage of DNA [6]. Bi-allelic loss of MutY homologue by itself is non oncogenic, but increases the risk of somatic G:C→T:A mutation at the APC locus [7]. To make things more complicated, the landscape of possible pathways has been newly enhanced by the discovery of the POLE and POLD1 germline mutations. Polymorphism of both mentioned genes significantly increases the somatic mutation rate and adenomagenesis by CIN-way [7]. Another fact worth mentioning regarding potentially targeted therapy, is the important route leading to transcription activation and consequently, tumorigenesis,. This route is the MAPK/mTOR pathway. Mutations of RAS, KRAS, BRAF, PTEN, STAT3 or PIK3CA can result in EGFR dependent tumorigenesis and progress [1, 2].

As about 95% of CRCs are sporadic in nature, it seems that crucial paths leading to a common endpoint are based on the Wnt-pathway, CIMP and deeply penetrating MSI. All aforementioned pathways, such as TP53,MAP, POLE/POLD1,MAPK/mTOR mutations, could often only be accompanying events. Additionally, some mutations like the SMAD family genes, especially SMAD4 with the imbalance of the TGF-β pathway, could result in a worse clinical outcome and higher risk of metastases [1, 2]. A compilation of potential various molecular events makes CRC one of the most genetically composite tumours. About 140 genes were classified as candidate cancer genes [1]. For prediction and also for effective treatment, the contribution of various molecular events, and especially their timing, are very important. If we assume that metastasis is caused for the most aggressive cells clones with additional mutations, we will diagnose real harmful potential of cancer with metastasis sample instead primary tumour sample. It could destroy some oncological paradigms in future.

### Goals

The study has been designed to present heterogeneous features and involved pathways within conventional colon adenomas. We have observed classical morphological patterns such as the usual tubular or villous architecture, but quite often they also present serrated foci. We aimed to evaluate a microsatellite stability status, β-catenin, BRAF V600E and p53 within classical polypoid colon adenomas. We have tried to explain the leading pathways, also in places with a serrated pattern.

## Material and methods

One-hundred and one (101) eligible cases of conventional polypoid colon adenomas were incorporated into the study. The participants of the Polish Colorectal Cancer Screening Program, supervised by the Polish Health Ministry, have been included to the ethnically homogenous (Caucasian) cohort over the period 2010–2015. The patients’ written consent have been obtained in the all cases. The patient’s approval-statement has possessed a clause concerning anonymous, scientific, not profit, using of all data collected during the Screening Program. An including criteria were stated as follows: complete resection of polyp and high grade epithelial dysplasia with the concurrent presence of low grade dysplasia according to the strict WHO criteria [8]. The pure serrated adenomas, sessile serrated polyps et hyperplastic polyps were excluded from the study. For the depiction of a comprehensive morphological pattern of adenomas with their heterogeneity, the classical immunohistochemistry method was used.

### Immunohistochemistry

In order to achieve our goals, an immunohistochemical examination of β-catenin, BRAF V600E, p53, MLH 1, MSH 2, MSH 6 and PMS 2 was performed, respectively. The details of used antibodies are presented in Table 1.

**Table 1:** The characteristics of used antibodies.

	Clone	Catalog Number	Dilution	Type of antibody	Manufacturer
Β-catenin	14	760–4244	Ready to use	Monoclonal mouse	Cell Marque
BRAF V600E	VE1	7904855	Ready to use	Monoclonal mouse	Ventana Medical Systems; Roche Group, Tucson, USA
p53	Bp-53-11	760–2542	Ready to use	Monoclonal mouse	Ventana Medical Systems; Roche Group, Tucson, USA
MLH 1	M1	790–4535	Ready to use	Monoclonal mouse	Ventana Medical Systems; Roche Group, Tucson, USA
MSH 2	G219-1129	760–4265	Ready to use	Monoclonal mouse	Cell Marque
MSH 6	44	790–4455	Ready to use	Monoclonal mouse	Ventana Medical Systems; Roche Group, Tucson, USA
PMS 2	EPR 3947	760–4541	Ready to use	Monoclonal rabbit	Cell Marque

The details connected with the previously analysed COX-2 within the same group were described in our previous publication [9].

The immunohistochemical assays were performed using the automated IHC/ISH slide staining system BenchMark XT (Ventana Medical Systems; Roche Group, Tucson, USA). After deparaffinisation and rehydration of the samples, the unmasking processes by CC1(Ventana Medical Systems; Roche Group, Tucson, USA), incubation with primary antibodies (time and temperature of both antigen retrieval and primary antibody incubation were in accordance with the manufacturer’s recommendations) and the further routine steps were performed. As a detection kit we used the Ventana ultra View Universal DAB Detection Kit and OptiView Detection Kit only for BRAF V600E (Ventana Medical Systems; Roche Group, Tucson, USA).

The cut-off expression criteria of the studied proteins were stated as:

β-catenin–any nuclear reaction as positive
p53- any strong nuclear reaction as positive
MLH-1, MSH2, MSH6, PMS2 –complete diffuse lack of nuclear reactions as positive MSI event
BRAFV600E –any level of cytoplasmic reaction as positive
COX-2 –strong cytoplasmic reaction as positive

### Statistical analysis

Categorical data were expressed as number and percentage distributions. Differences in proportions were assessed using Fisher’s exact test. Quantitative data were characterized by means and standard deviations and the unpaired t-test or Mann-Whitney *U* test was used for comparisons between groups, as appropriate. Two tailed p-value < 0.05 was considered to be statistically significant.

All computations were performed using the statistical package R, version 3.1.2 (R Core Team (2014). R: A language and environment for statistical computing. R Foundation for Statistical Computing, Vienna, Austria.URL http://www.R-project.org/.)

### Ethical correctness statement

This study with the use of human tissue was in accordance with the ethical standards of the declaration of Helsinki with its latest revision in 2004. Additionally, the study was approved by the Ethics Commission of the Faculty of the Medicine and Health Science, Jan Kochanowski University in Kielce, Poland (Decision No. 10/2016).

## Results

The general patient and tumour characteristics have been presented in Table 2. Classical tubular adenomas comprised 57%, mixed tubule-villous 40%, but mixed tubulo-serrated, according to WHO guidelines, about 4% [8].

**Table 2:** The general characteristics of patients and polyps with frequency ratio of evaluated items.

	n = 101
Age/years/	66.4 (±9.8st.dev) 40–90[min-max]
Gender	
Female	32 (31.7%)
Male	69 (68.3%)
Polyp size (mm)	16.3 (±8.7st.dev) 5–60 [min-max]
Location	
Right colon	13 (12.9%)
Left colon	62 (61.4%)
Rectum	26 (25.7%)
Histopathological type of adenomas according to WHO	
Tubular adenoma	57 (56.4%)
Mixed tubular and serrated adenoma	4 (4.0%)
Tubulovillous adenoma	40 (39.6%)
Presence of microserrated foci	20 (19.8%)
Occurring frequency of targeted disturbances	
COX-2 overexpression	60 (59.4%)
β-catenin nuclear expression	71 (70.3%)
p53 overexpression -within high grade dysplasia foci	24 (23.8%)
p53 overexpression -within low grade dysplasia foci	6 (5.9%)
MSI events–generally	10 (9.9%)
lack of MSH-2 and MSH-6	2(2.0%)
lack of MSH-2, MSH-6, PMS-2, MLH-1	1(1.0%)
lack of MSH-2, PMS-2, MLH-1	2(2.0%)
lack only MSH-6	3(3.0%)
lack only PMS-2	2(2.0%)

**Fig 1:**
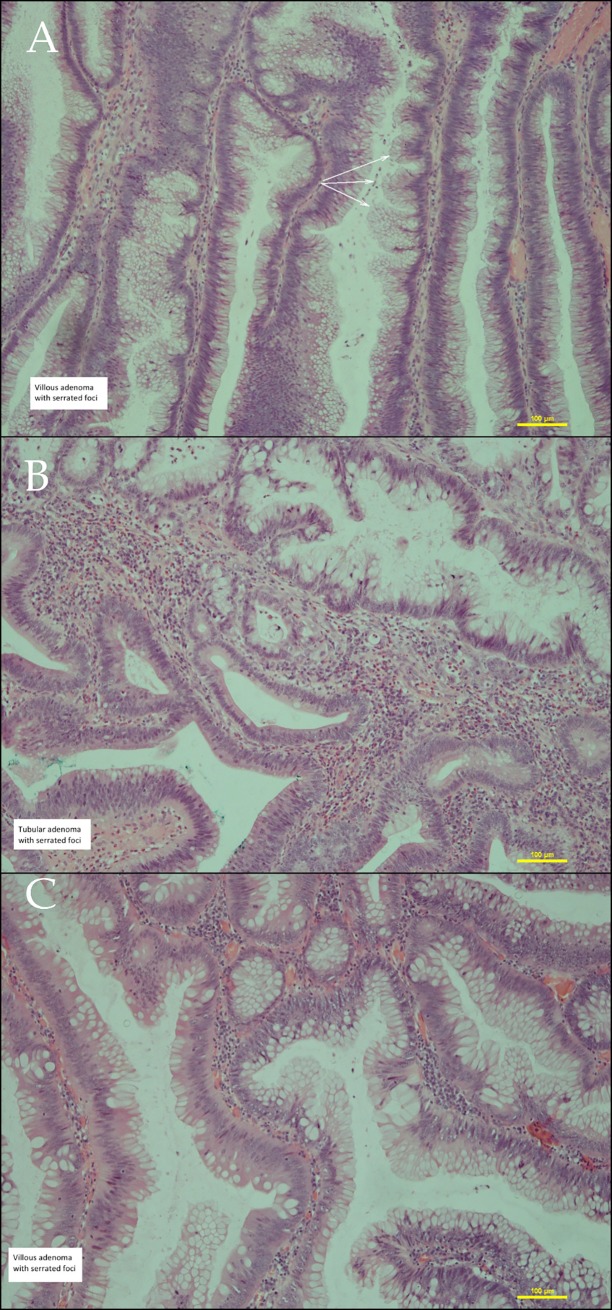
Various patterns of micro-serration within classical adenomas. (A-C) Note, there are hyperplastic epithelial folding with saw-teeth appearance without nuclear stratification and cytoplasmic eosinophilia. (A) Epithelial folding into usual villous adenoma. (B-C) The outstanding micro-serration into tubular adenoma. H&E stain.

The presence of serrated foci has been documented within 20% of adenomas (Fig 1). As presented in Table 2, the most frequent genetic anomalies were Wnt-pathway disturbances unveiled by a 70% ratio nuclear expression of β-catenin. Not surprisingly, the contribution of the p53 overexpression was growing in line with the progress of the dysplastic level, finally achieving a 24% share. Our main target was the analysis of MSI/MSS status in traditional colon polyps. Here, we noted 10 cases with various levels of disturbances within MSI driving genes. It is worth highlighting the five MSI-H cases where four had a left-sided location and typical traditional histopathological features. (Table 2) It mirrors the precursor lesions with the MSI pathway which are present fairly often also in the left colon which then could transform to conventional cancer. Consequently, the morphological appearance of cancer, without the typical histopathological features MSI carrying cancers, mask the true driving pathway.

**Fig 2:**
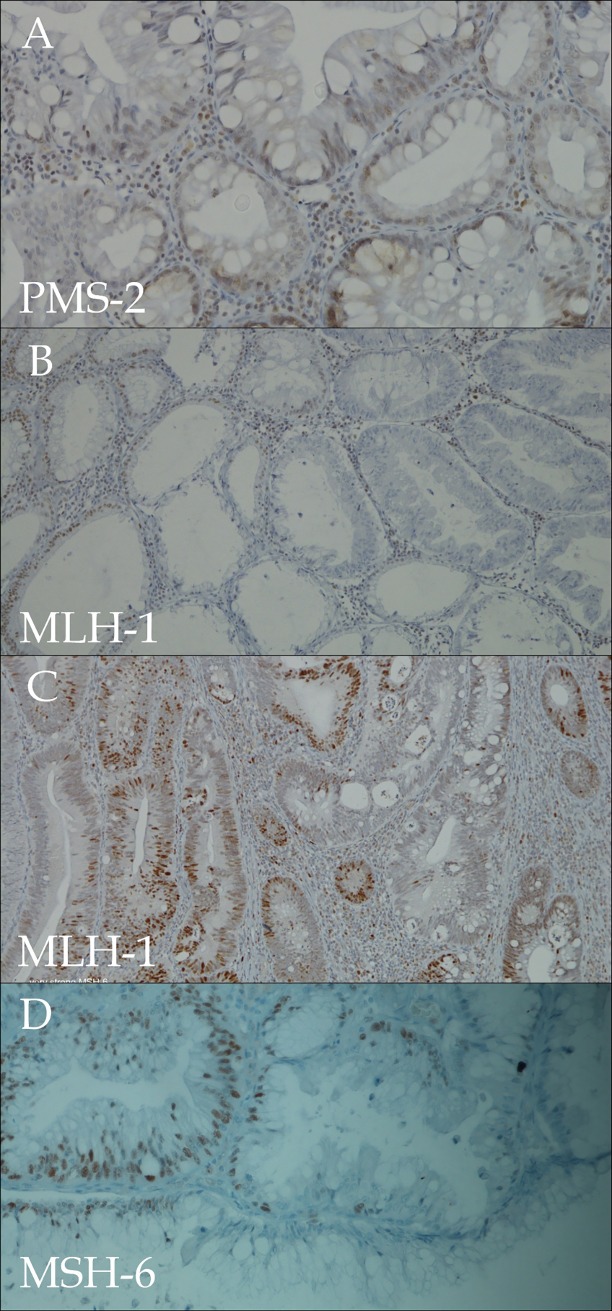
Compilation of microserrated foci with loss of microsatellite stability. (A-D) The pictures show patchy heterogeneity in scope MSI while the majority of adenomas were MSS. Note, here is evident epithelial micro-serration with concurrent lack of expression of targeted proteins.

As has been presented in Table 3, we have not observed dependencies in the scope of MSI events generally and separately with respect to age, sex, polyp size, location or presence of serrated foci. Comprehensive statistical analysis showed a connection of MLH-1 absence with a tubule-villous type of polyp (p = 0.033) but the meaning of that should be restricted due to the small figures events. The significant differences have been noted in the scope of the presence of micro-serrated foci with polyp type. It has been noted that there is a positive correlation with typical tubular adenomas (p = 0.001). The analysis of p53 overexpression has revealed differences with gender (p = 0.043) and also with β-catenin. (p = 0.002, not presented in the table). The florid heterogeneity also in range of TP53 disturbances have been additionally presented in S1 Fig and S2 Fig. The analysis of BRAF V600E was performed in all cases with micro-serrated foci and in all cases with an MSI event. All of the assays were negative. A confirmation of MSI needs a clear immunohistochemical reaction with a diffused absence of targeted protein. This results in doubts when presented with analyses of cases with evident heterogeneity. As depicted in Fig 2 and also S3 Fig, micro-serrated foci often were MSI, while the majority of the polyps were MSS.

**Table 3:** A consolidated presentation of achieved results with the statistical notes.

	PMS-2		MLH-1		MSH-2		MSH-6		β-catenin		Presence of serrated foci		p53		COX-2		BRAF
	lack	p	lack	p	lack	p	lack	p	posiive	p	yes	p	positive	p		p	
Number of events	5		2		6		5		71		20		24		60		0
Age* (years)	68.0 (±13.6)	0.52	76.0 (±9.9)	-	65.5 (±15.6)	0.94	61.0 (±7.6)	0.20	66.0 (±9.9)	0.49	68.7 (±11.3)	0.14	63.0 (±11.5)	0.098	66.5 (±9.6)	0.76	n/a**
Gender		1.0		0.54		1.00		1.0		0.49		0.06		0.04		0.39	
Female	1 (20.0%)		1 (50.0%)		2 (33.3%)		1 (20.0%)		21 (29.6%)		10 (50.0%)		12 (50.0%)		17 (28.3%)		
Male	4 (80.0%)		1 50.0%)		4 (66.7%)		4 (80.0%)		50 (70.4%)		10 (50.0%)		12 (50.0%)		43 (71.7%)		
Polyp size* (mm)	20.8 (±17.2)	0.77	35.0 (±21.2)	-	20.5 (±15.3)	0.62	17.2 (±18.4)	0.22	15.6 (±6.7)	0.90	16.2 (±5.7)	0.46	13.7 (±5.8)	0.11	17.2 (±9.3)	0.17	
Location		0.40		0.31		1.0		1.0		0.17		0.94		0.008		0.80	
Left colon	4 (80.0%)		150.0%)		4 (66.7%)		4 (80.0%)		41 (57.7%)		12 (60.0%)		11 (45.8%)		36 (60.0%)		
Rectum	0 (0.0%)		0 (0.0%)		1 (16.7%)		1 (20.0%)		22 (31.0%)		5 (25.0%)		12 (50.0%)		15 (25.0%)		
Right colon	1 (20.0%)		150.0%)		1 (16.7%)		0 (0.0%)		8 (11.3%)		3 (15.0%)		1 (4.2%)		9 (15.0%)		
Serrated foci		1.00		0.36		0.34		0.58		0.59				0.56		1.0	
no	4 (80.0%)		1 (50.0%)		4 (66.7%)		5 (100.0%)		58 (81.7%)				18 (75.0%)		48 (80.0%)		
yes	1 (20.0%)		1 (50.0%)		2 (33.3%)		0 (0.0%)		13 (18.3%)				6 (25.0%)		12 (20.0%)		
Adenoma type		0.07		0.03		0.16		0.71		1.00		0.001		0.12		0.77	
Tubular	1 (20.0%)		0 (0.0%)		2 (33.3%)		4 (80.0%)		40 (56.3%)		11 (55.0%)		16 (66.7%)		33 (55.0%)		
Mixed tubulo-serrated	1 (20.0%)		1 (50.0%)		1 (16.7%)		0 (0.0%)		3 (4.2%)		4 (20.0%)		2 (8.3%)		2 (3.3%)		
Tubulovillous	3 (60.0%)		1 (50.0%)		3 (50.0%)		1 (20.0%)		28 (39.4%)		5 (25.0%)		6 (25.0%)		25 (41.7%)		

*Age and polyp size are presented as mean (± standard deviation).

** not applicable.

## Discussion

CRC is still one of the most investigated cancers. It is a consequence of its frequency and very vast landscape of driving pathways. The progress of science and long-term observations of clinical outcomes resulted in splitting CRC to left-, right-sided and rectal located tumors. All this has had underlying molecular alterations which has been explained step by step to the present day. MSI cancers are routinely perceived as right colon cancers with a mucinous or medullary component, often with prominent lymphoid infiltration and rarer granulomatous Crohn-like response. Notwithstanding, these cancers are often connected with a higher pT or grade level, paradoxically the clinical outcome is better. Additionally there are contradictory researches about the overall survival (OS) rate in light of 5-fluorouracil based chemotherapy and the predictive value of MSI status in planning adjuvant therapy [10–12]. At present, neoadjuvant chemotherapy schemes such as IFL, FOLFOX or fluorouracil-bevacizumab are used without clear guidelines based on molecular signaling paths[13]. Currently, we dispose the blocking EGFR dependent MAPK/mTOR path by cetuximab and VEGF dependent signaling by bevacizumab. The high cost of personalized treatment imposes confirmation of EGFR expression and RAS/KRAS wild-type status before treatment, unfortunately it is often second-line treatment [14]. All that which has been stated is beyond question, but the next question is how to manage tumours of composite origin or florid heterogeneity. Moreover, screening for an underlying CRC pathway is needed. Routinely, KRAS status is based on the analysis of the usual FFPE tumour sample. Considering tumour heterogeneity and molecular timing issues, we state a thesis that lymph node metastasis might be a better choice for searching for a target for only one available targeted chemotherapy.

Lynch’s paradigm of the ‘non-polyposis’ appearance of MSI cancers resulted in a compartmentalization of all the rest of polypoid lesions as MSI independent. The description of a serrated pathway, where hypermetylation of the MLH-1 promoter often occurs, again emphasized the possibility of MSI within various polypoid lesions. This led us to the inclination of analysis of MSI events within traditional adenomas. Our results confirmed the leading role of the Wnt-path with a high rate of TP53 mutations, but which simultaneously presented a 10% frequency of MSI events. The aforementioned five cases with typical tubular or tubulo-villous features carried MSI-H status without β-catenin and p53 expression unveil a possibility of MSI driven path also in conventional adenomas. Moreover, one polyp had a rectal location and a further three polyps were located in the left-side of the colon. This depicts MSI as the main driving pathway also within conventional adenomas, and consequently cancer. Despite no correlations between the presence of microserreted foci with MSI events we observed typical for CRC heterogeneity also in areas of serrations. (Table 3) (Fig 2 and S3 Fig). This could illustrate tumour heterogeneity, but on the other hand, concurrent different pathways could have affected the polyp. A mosaic of underlying pathways within typical adenomas naturally results in a morphological pattern of consequent cancer. Of note, our cohort presents a 17% frequency of serration in the left colon and rectal adenomas, and besides no statistical correlation depicts a high level of heterogeneity. We observed in the serrated foci a typical hyperplastic, with saw and teeth appearance, epithelium, without prominent nuclear stratification and cytoplasmic eosinophilia what might suggested a true serrated pathway. Additionally, the lack of p53, β-catenin and BRAFV600E expression points to MSI disturbances as the main driving pathway. Obviously, the explanation of the basis of these events, inherited mutation or gene promoter hypermetylation, calls for the microdissection method. In our daily work, we have often observed various, often with a mucinous type, histopathological types of adenocarcinoma into a single tumour. In fact, a cocktail of varied histopathological types illustrates many pathways involved and simultaneously acquired additional molecular hits. Setting aside CRC heterogeneity’s meaning, Kolos et al. [15] reported the loss of MSH-2 with a 2% frequency within left-sided typical CRCs, which should be taken into consideration in unequivocal discrimination of left- and right side CRC based only on a morphological pattern.

Hard evidence shows the overwhelming dominance of the Wnt-pathway in adenomagenesis. A recent study by Muto et al. [16] presented the silencing of the AXIN-2 gene due to its hypermetylation. The involvement of hypermetylation is becoming more and more interesting. It concerns the genes Wnt-pathway and other, for instance MLH-1 as well. MLH-1 silencing results in secondary MSI and AXIN-2 disturbances leading to β-catenin downstream and cell proliferation. Other Wnt antagonists like SFRP1, SFRP2, SFRP5, DKK2,WIF1 and also c-MYC should be hypermetylated and result in β-catenin action as well [17]. This alternative to typical frameshift mutation pathway impacts the same endpoint effects but could change the tumour appearance, because methylation is often connected to the presence of serration. For many years it was believed that the right sided typical SSA are driven by CIMP and counteractively to conventional adenomas by CIN, especially due to the APC gene’s frameshift mutation. A recent study by Li et al. [18] discredits the view regarding the lack of contribution of the Wnt-pathway within serrated adenomas. Notably, their study showed close to a 40% N-terminus β-catenin expression within SSA and hyperplastic polyps [18]. The meaning of N-terminus contribution instead of C-terminus involved in the typical Wnt-path is unclear, but it makes room for follow-up research. The MSI and CIN-pathways are only seemingly independent. Jorissen et al. presented the protective influence of the wild-type APC in MSS, both proximal and distal CRC. No dependencies were observed in the case of MSI, moreover the influence was stronger than KRAS, BRAF, PIK3CA, TP53 and CpG methylation as well. The poor clinical outcome of right-sided wild-type APC/MSS cases authors joined with serration-pathway rather [19].

The absence of BRAFV600E mutation with MSI is considered an inherited mutation and rather excludes Lynch syndrome [11, 12]. The study by Wojcik et al. based on ethnically similar cohort showed a 3,7% frequency BRAF mutation. No differences with location and MSS/MSI were observed [20]. Our results with lack the BRAF V600E expression confirm the low frequency of BRAF mutation and suggest that a timing of mutations is in a late phase of tumorigenesis.

An experimental study by Lemieux et al. unveiled the potential impact of BRAF mutation through kinase MAK1 action. The authors stated a thesis that MAK1 induced a phosphorylation of transcription factors that could also enhance the quantity of β-catenin and thereby start a canonical Wnt-path without APC loss or attenuation [21].

In summary, CRCs are largely a heterogenic group of cancers. The compartmentalization of CRC as ‘colorectal cancer’ is general, when in fact they represent varied cancers driven by different pathways with different clinical outcomes, various responses to chemotherapy and finally, often with similar histopathological features. In the age of molecular analyses and personalized treatment, there is an essential need to identify the key CRC subtypes based on molecular fundamentals.

## Conclusions

The main goal of this study is the analysis of MSI frequency and its contribution to conventional colon adenomas. We presented typical for CRC heterogeneity, also in the scope of MSS/MSI status. The results generally indicate a nearly 10% frequency of MSI events. Indeed, 5% of usual adenomas is able to carry MSI-H. These notes illustrate a larger contribution of MSI than had been presumed before.

## Supporting information

S1 Fig The p53 monotonous overexpression. This case illustrates a strong TP 53 contribution with typical superficial location and crypts sparing.
(TIF)

S2 Fig The florid heterogeneity of p53 into usual adenoma. Note, left side of the picture includes foci with micro-serration and lack of p53 overexpression while right side is substantial in TP53 disturbances.
(TIF)

S3 Fig The focal lack of MLH-1. A case of common Wnt-path with outstanding micro-serration and focal lack of MLH-1 what could suggests a focal MLH-1 hypermetylation and various pathways involved.
(TIF)
