# Supplementary figures and images for: Conventional colon adenomas harbor various disturbances in microsatellite stability and contain micro-serrated foci with microsatellite instability
Source: PLoS One. 2017 Feb 24;12(2):e0172381. doi: 10.1371/journal.pone.0172381 (PMC5325232; doi:10.1371/journal.pone.0172381)

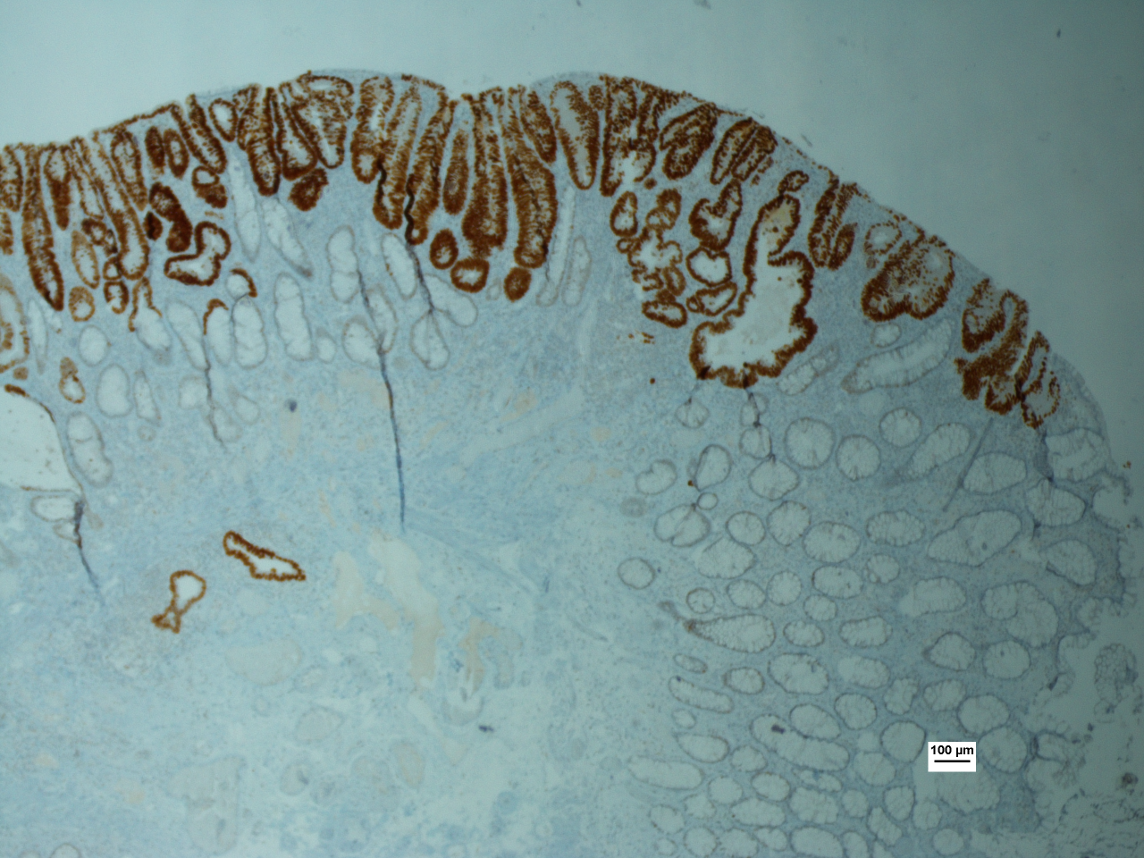

Supplement: S1 Fig — This case illustrates a strong TP 53 contribution with typical superficial location and crypts sparing. (TIF) [file pone.0172381.s001.tif]

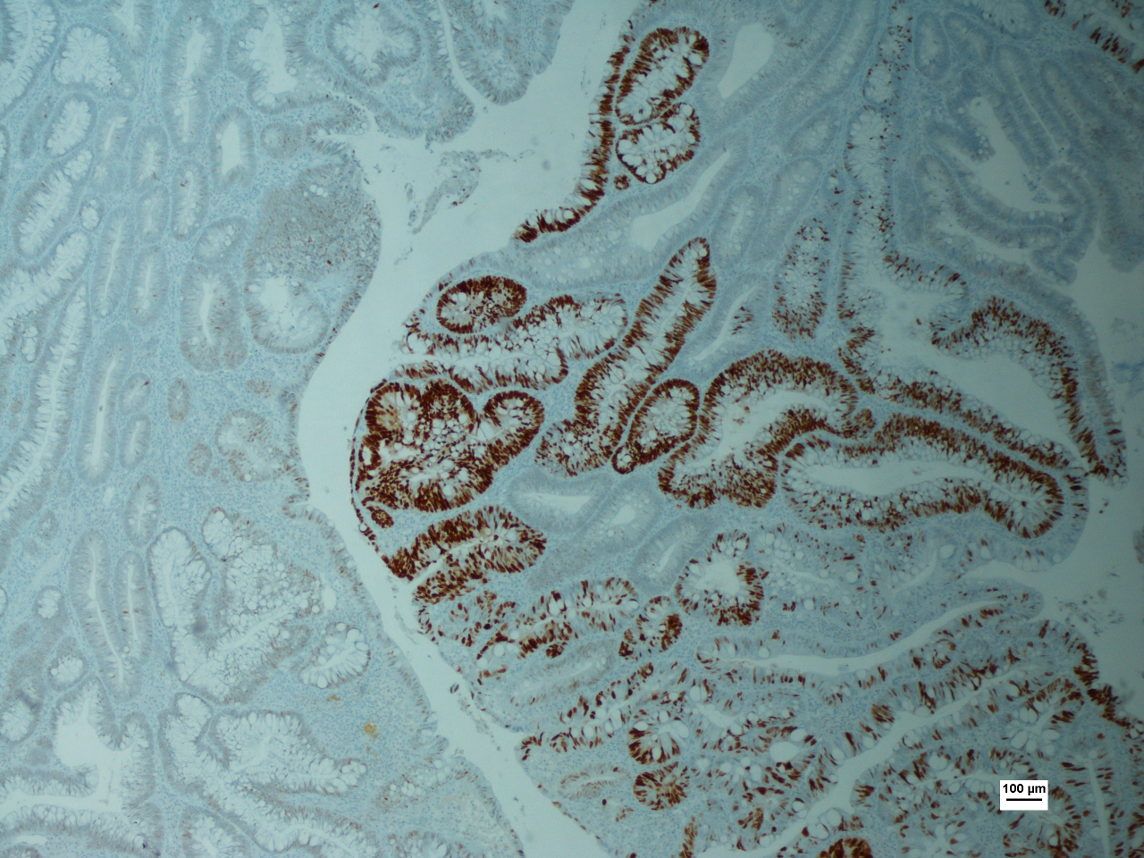

Supplement: S2 Fig — Note, left side of the picture includes foci with micro-serration and lack of p53 overexpression while right side is substantial in TP53 disturbances. (TIF) [file pone.0172381.s002.tif]

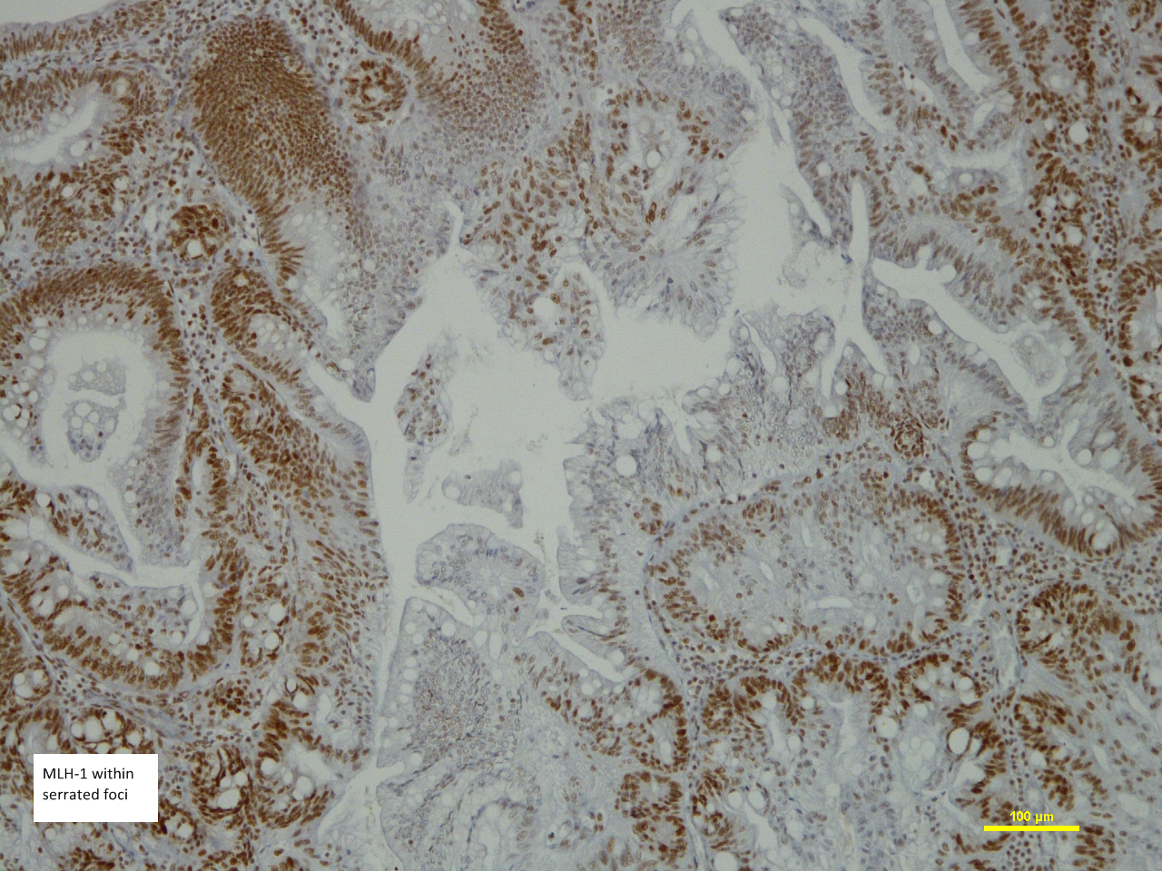

Supplement: S3 Fig — A case of common Wnt-path with outstanding micro-serration and focal lack of MLH-1 what could suggests a focal MLH-1 hypermetylation and various pathways involved. (TIF) [file pone.0172381.s003.tif]
